# NQO1 Deficiency Aggravates Renal Injury by Dysregulating Vps34/ATG14L Complex during Autophagy Initiation in Diabetic Nephropathy

**DOI:** 10.3390/antiox10020333

**Published:** 2021-02-23

**Authors:** Geum-Lan Hong, Kyung-Hyun Kim, Chul-Ho Lee, Tae-Won Kim, Ju-Young Jung

**Affiliations:** 1 Department of Veterinary Medicine & Institute of Veterinary Science, Chungnam National University, Daejeon 34134, Korea; ghdrmafks@o.cnu.ac.kr (G.-L.H.); kyunghyun@cnu.ac.kr (K.-H.K.); taewonkim@cnu.ac.kr (T.-W.K.); 2 Laboratory Animal Resource Center Korea Research Institute of Bioscience and Biotechnology, Daejeon 34141, Korea; chullee@kribb.re.kr

**Keywords:** diabetic nephropathy, NQO1, autophagy, renal injury

## Abstract

Diabetic nephropathy (DN) is one of the causes of end-stage renal failure, featuring renal fibrosis. However, autophagy, a vital process for intracellular homeostasis, can counteract renal fibrosis. Moreover, NAD(P)H: quinone dehydrogenase 1 (NQO1) modulates the ratios of reduced/oxidized nicotinamide nucleotides, exerting a cytoprotective function. Here, to examine the role of *NQO1* genes in DN progression, the levels of autophagy-related proteins and pro-fibrotic markers were assessed in silencing or overexpression of *NQO1* in human proximal tubular cells (HK2), and C57BL/6 (wild-type) and *Nqo1 knockout* (KO) mice injected to streptozotocin (50 mg/kg). NQO1 deficiency impaired the autophagy process by suppressing basal expression of ClassⅢ PI 3-kinase (Vps34) and autophagy-related (ATG)14L and inducing the expressions of transforming growth factor beta (TGF-β1), Smad3, and matrix metallopeptidase9 (MMP9) in high-glucose (HG) -treated HK2 cells. Meanwhile, *NQO1* overexpression increased the expression of Vps34 and ATG14L, while, reducing TGF-β1, Smad3 and MMP9 expression. *In vivo*, the expression of Vps34 and ATG14L were suppressed in *Nqo1* KO mice indicating aggravated glomerular changes and interstitial fibrosis. Therefore, *NQO1* deficiency dysregulated autophagy initiation in HK2 cells, with consequent worsened renal cell damage under HG condition. Moreover, STZ-treated *Nqo1* KO mice showed that *NQO1* deficiency aggravated renal fibrosis by dysregulating autophagy.

## 1. Introduction

Diabetic nephropathy (DN) typically evolves by gradual progression from microalbuminuria to proteinuria, usually about 15 years after the onset of diabetes [1]. Diabetic kidney disease is characterized by glomerular hyperfiltration and declining glomerular filtration rate, ultimately leading to end-stage renal disease and accounting for nearly 50% of all cases of this pathology [2,3,4]. Alterations in the structure of kidney during DN are thickening of glomerular basement membrane and of the tubular basement membrane, mesangial matrix expansion, hyalinosis and tubulointerstitial fibrosis (TIF) [5]. Glomerulosclerosis, TIF, and activation of alpha-smooth muscle actin (α-SMA) positive myofibroblast are renal fibrotic changes, among which TIF represents the key pathological alteration of end-stage renal disease [6,7].

Kidney fibrosis is a chronic and progressive process and represents the common final outcome of nearly all chronic and progressive nephropathies. Fibrotic matrix deposition disrupts organ structure, reduces blood supply and induces organ failure [8]. Transforming growth factor-β (TGF-β), a master regulator of myofibroblast differentiation [9,10,11], is the primary factor inducing fibrosis in most forms of chronic kidney disease [12]. Indeed, TGF-β1 drives glomerular and tubulointerstitial fibrosis [13,14,15] and regulates many biological processes including differentiation, cell proliferation, apoptosis, and autophagy [16,17]. TGF-β1 induces renal fibrosis via activation of a Smad-dependent pathway through the phosphorylation and activation of Smad3 [18], resulting in stimulation of myofibroblasts, excessive production of extracellular matrix (ECM) and inhibition of ECM degradation [12,19]. In fact, Smad3 directly binds to gene promoters to induce the transcription of pro-fibrotic molecules, such as α-SMA [12].

Autophagy is a dynamic process by which intracellular damaged macromolecules and organelles are degraded and recycled for the synthesis of new cellular components [20]. Basal autophagy in the kidney acts as a quality control system and is vital for cellular remodeling and intracellular homeostasis [21,22]. Therefore, dysregulation or failure of the autophagy results in various human pathologies, including cancer, neurodegenerative diseases, chronic inflammatory diseases, etc. [23,24,25]. Various stresses, including oxidative stress, hypoxia, and nutrient and energy depletion can activate autophagy in renal tubular epithelial cells and podocytes. The role of autophagy in renoprotection was studied in unilateral ureteral obstruction models in which the inhibition of autophagy enhanced tubular apoptosis and interstitial fibrosis, and increased the deposition of collagen through the upregulation of TGF-β1 [26,27,28]. Recently, De Pascalis A et al. reported that sodium-glucose cotransporter 2 inhibitors (SGLTi), a new class of antidiabetic drugs, leads to increased autophagy and enhances AMPK/SIRT1 signaling underpinning the protective effects of DN [29]. Moreover, a recent study showed that displaying streptozotocin (STZ)-induced DN, micro RNA(miR)-22 upregulation was associated with increased fibrosis and suppression of autophagy whereas traditional Chinese medicine could reduce fibrosis by promoting autophagy [30,31].

NAD(P)H:quinone dehydrogenase 1 (NQO1) is a homodimeric flavoprotein that catalyzes the two-electron reduction of quinones by using either NADPH or NADH as hydride donor [32]. Under oxidative stress, the expression of NQO1 is triggered by the Keap1/NRF2/ARE pathway and modulates the ratios of reduced/oxidized nicotinamide nucleotides, thereby exerting multiple cytoprotective functions, such as microtubule stabilization and increased expressions of other tumor suppressor proteins [32,33]. Consistently, several lines of evidence have revealed that NQO1 disruption enhances susceptibility to oxidative stress [34,35]. Oxidative stress has been recognized as a key factor in the pathogenesis and progression of DN; also, various symptoms including hyperglycemia, accumulation of reactive oxygen species, high arterial pressure, and expression of inflammatory markers indicate the presence of oxidative stress in DN [36].

In the present study, we examined the role of NQO1 in the modulation of autophagy during STZ-induced DN. Furthermore, we studied renal damage progression in *Nqo1 knockout*, STZ-induced DN model mice. As *NQO1* mutation modulated the autophagy process, this gene may play an important role in kidney protection against DN.

## 2. Materials and Methods

### 2.1. Cell Culture and Treatment

Human cortex proximal tubular cells line (HK2) was perched from Korean Cell Line Bank, Seoul, Korea. HK2 cells were grown in RPMI-1640 (RPMI; Gibco, MA, USA) media supplemented with 10% fetal bovine serum (Gibco), and 1% penicillin and streptomycin (Gibco) at 37 °C in a 5% CO2 atmosphere. The medium was replaced every 48 h. After seeding an equal density of 3 × 105 cells/well in RPMI in six-well plates, HK2 cells were starved for 24 h. HK2 cells treated with normal (5.5 mM), middle (10 mM) and high level of glucose (25 mM, Sigma-Aldrich, MO, USA) were harvested 24 h post stimulation. For autophagy inhibition studies, the cells were treated with 3-methyladenine (3-MA, 5 mM) for 3 h or rapamycin (100 nM) for 24 h before harvested.

### 2.2. Modulation of NQO1 Expression In Vitro

siRNA was used to explore the cellular effects of NQO1 silencing. HK2 cells were seeded on six-well plate (5 × 10^5^/well) and incubated for 40 h with scrambled siControl or siRNA targeting human NQO1 (5’-AAACCAGCCUUUCAGAAUGGCUGGC-3’, 20 nM; Invitrogen, CA, USA) together with a transfection reagent (Invitrogen). After incubation, the cells were treated with high glucose as described earlier and subjected to western blot analysis.

For NQO1 overexpression, cells were seeded at a concentration of 5 × 10^5^/well in six-well plates. Two μg of NQO1 overexpression plasmids (Strep-tag pEXPR-IBA105-NQO1, AH005427.2) produced by Dr. Jong-Soo Lee Chungnam National University, Daejeon, Korea) were transfected with 2 μg by using Lipofectamine 2000 (Invitrogen) as described in the manufacture’s protocol. After incubation, the cells were treated with high glucose as described earlier and harvested for analysis.

### 2.3. Immunofluorescence

Immunofluorescence was performed according to method in use in the laboratory [34]. Cells were fixed with absolute alcohol, washed, and, blocked with 3% bovine serum albumin in PBS for 30 min. The cells were then incubated overnight with anti-Vps34 (1:500; Abcam, Cambrige, UK), anti-ATG14L (1:500; Cell Signaling Technology, Danvers, MA, USA), anti-collagen (1:500;Novus Biologicals, CO, USA) and anti-fibronectin (1:500; abcam) antibodies. The cells were washed with blocking solution for 30 min and then incubated for 1 h with goat anti-rabbit Alexa Fluor^®^ 594 (IgG) secondary antibody (Life Technologies, CA, USA) diluted in blocking solution. Cells were washed with PBS and then mounted with ProLongTM Gold Antifade Mountant with DAPI (Invitrogen). Cells were observed with the Nikon 80i microscope (Nikon, Tokyo, Japan) under the automatical exposure time for each wavelength or Zeiss LSM 880 with Airyscan confocal microscopy (Zeiss, Jena, Germany) wavelengths of 488 and 592 nm. Images were captured with the DP Controller software.

### 2.4. Animal Study

All animal experimental protocols were approved by the Institutional Animal Ethic Committee of the Chungnam National University (CNU-01107). The research was conducted in accordance with the Guide for the Care and Use of Laboratory Animals of the National Institutes of Health. C57BL/6 Nqo1 knockout (KO) mice were donated by Dr. Chul-Ho Lee (Korea Research Institute of Bioscience and Biotechnology, Daejeon, Korea). For controls, non-transgenic age-matching mice were purchased from Orient Bio (Gyeonggi-do, Korea). All animals were acclimated under specific pathogen-free conditions in individual cages with controlled temperature (22 ± 2 °C) and humidity (55 ± 5%) under a 12 h light cycle for 1 week before the experiment and fed with standard chow and water ad libitum.

The animals were divided into four groups (*n* = 5); wild-type controls (treated with phosphate buffered saline (PBS)); wild-type mice treated with STZ; Nqo1 KO control (treated with PBS); and Nqo1 KO mice treated with STZ. To induce DN, mice in STZ-treated groups were injected intraperitoneally with 50 mg/kg STZ daily for five days. After three days, the blood glucose levels were measured with a glucometer (GlucoNavii, SD Biosensor, Gyeonggi-do, Korea), and mice displaying a level of blood glucose > 300 mg/dL were considered diabetic and used in this experiment. 12 weeks later, at the end of experiment, the mice were fasted and euthanized with CO_2_.

### 2.5. Measurement of Metabolic and Blood Parameter

Water intake and urination were measured using metabolic cages and blood was collected from the posterior venous vein. The collected blood samples were centrifuged for 15 min at 3000× *g*, and the serum was stored at −80 °C for subsequent analysis. The levels of blood urea nitrogen (BUN) and creatinine of blood samples were measured using a DRY-CHEM instrument (Fujifilm, Tokyo, Japan) according to the manufacturer’s instructions. The levels of glycosylated hemoglobin A1c (HbA1c) were determined using a commercial ELISA kit (Rat HbA1c ELISA Kit, MyBiosource (San., CA, USA) according to the manufacturer’s protocol.

### 2.6. Western Blot

Collected cells and frozen tissue samples were quantified as described previously [37]. The membrane was blocked with 5% skim milk in 1× PBS with 20% Tween-20 (PBS-T) for 1–2 h and incubated with primary antibodies (S1). After incubation, horseradish peroxidase-conjugated anti-mouse (1:5000, AbFrontier, Seoul, Korea), Goat anti-rabbit (1:5000, AbFrontier), and donkey anti-goat (1:5000, Santa Cruz, Dallas, TX, USA) IgG secondary antibodies were used to detect each proteins. Proteins were visualized with an enhanced chemiluminescence (ECL) detection kit (Amersham Pharmacia Biotech, Little Chalfont, UK) and quantified using the Image Lab Software (Bio-Rad) or Image J software (Image J v1.46a; NIH, USA).

### 2.7. Histopathological Analysis

Kidneys were fixed immediately in a 10% neutral buffered formalin solution (Sigma) and embedded in paraffin. Blocks were cut into 5 μm-thick sections which were then subjected to hematoxylin and eosin (H&E), periodic acid-Schiff (PAS), and Sirius red staining as per manufacturer’s protocols. Stained tissues were examined by light microscopy (Nikon ECLIPSE Ni-U, Tokyo, Japan) at a 400× magnification. The status of tissues was graded from 10 random images for each specimen in a blinded manner according to the international pathologic classification of DN lesions into glomerular and interstitial fibrosis and tubular atrophy (IFTA) [2]. The quantitative analysis of the area of renal fibrosis by Sirius red staining was measured form 10 random images for each specimen in blinded manner.

### 2.8. Statistical Analysis

All experiments were conducted in double blind. Results were randomly selected and expressed as mean ± SEM of triplicate experiments. GraphPad Prism version8.0 (GraphPad Software, La Jolla, CA, USA) was used for data analysis. The Mann-Whitney U test for non-parameteric data was used to assess the differences between two groups. For comparisons among multiple groups, one-way analysis of variance (ANOVA) was used, followed by post-hoc Tukey HSD test when relevant. A *p* value of < 0.05 was considered as a threshold for statistic significance.

## 3. Results

### 3.1. Expression of Autophagy and Fibrosis-Related Proteins in High-Glucose-Treated in HK2 Cells

**Figure 1:**
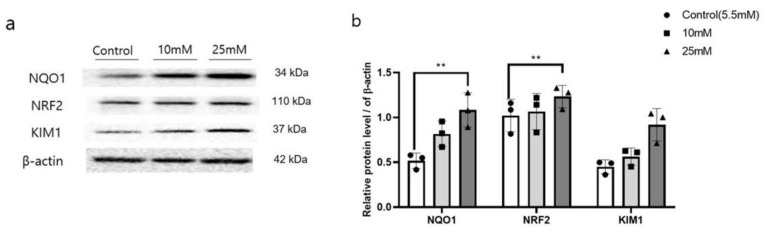
Increased autophagy related protein and kidney damage accompanied by enhanced expression of *NQO1* in high glucose treated HK2 cells. Cells were harvested 24 h after high glucose (10 mM, 25 mM) treatment, and total cell lysates were subjected to Western blotting. Band intensities of NQO1, NRF2 and KIM1 (**a**), were normalized to those of β-actin (**b**). Values are expressed as means ± SEM of triplicate experiments. ** *p* < 0.01, compared with control cells.

The expressions of NQO1 and NRF related to oxidative stress, was significantly enhanced (*p* < 0.05), together with the expression of kidney injury molecular 1 (KIM1), in HK2 cells treated with high glucose (HG, 25 mM) (Figure 1a). After confirming this result, the expressions of autophagy and fibrosis-related proteins was assessed in HK2 cells (S2, 3).

### 3.2. Inhibition of Autophagy Increased Renal Fibrosis-Related Proteins in HG-Treated HK2 Cells

**Figure 2:**
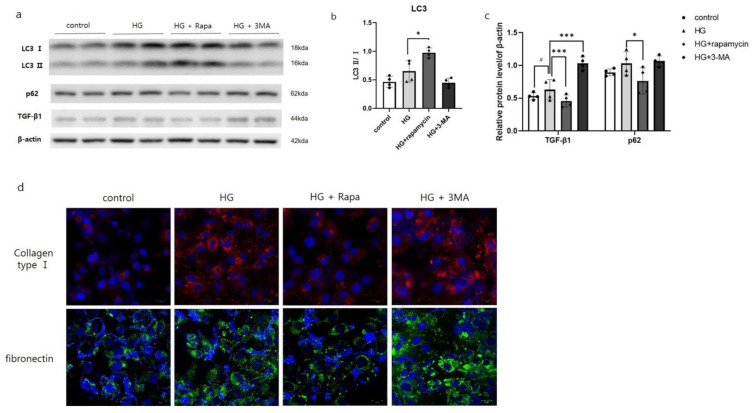
Autophagy inhibition increased renal fibrosis-related proteins in HG-treated HK2 cells. Cells were harvested 24 after high glucose (25 mM). Cell was treated with rapamycin (100 nM) for 24 h or 3-MA (5 mM) for 3 h before harvesting. Total cell lysates were subjected to western blotting. Conversion ratio of LC3Ⅱ/Ⅰ (**b**). The band intensities of p62, TGF-β1 were normalized to those of β-actin (**a**,**c**). Values are expressed as means ± SEM of triplicate experiments. ^#^ *p* < 0.01, compared with control cells, *** *p* < 0.001 and * *p* < 0.01, compared with HG cells. Cell nuclei were visualized by 6-diamino-2 phenylindole (DAPI; Blue) and the expression of collagen (red) and fibronectin (green) were visualized by Zeiss LSM 880 with Airyscan confocal microscopy at 400× magnification (scale bar = 20 μm) (**d**).

To assess functional significance of autophagy in HG-treated HK2 cells, 3-MA or rapamycin was co-treated with HG. The expression of LC3-Ⅱ was reduced in HK2 cells with HG and 3-MA co-treatment compared to HG-treated HK2 cells. On the other hand, the expression of LC3-Ⅱ was increased and p62 expression was decreased in HK2 cells co-treated with HG and rapamycin, mTOR inhibitor. Interestingly, the expression of TGF-β1 was increased in HG-treated HK2 cells. After HG and 3-MA co-treatment, the expression of TGF-β1 was reduced while increased in HG and rapamycin co-treatment compared to HG-treated HK2 cells (Figure 2a–c). Along with the results of TGF- β1, immunofluorescence staining results showed that collagen and fibronectin deposition was greatly increased in HG and 3-MA co-treatment and decreased in HG and rapamycin co-treatment compared to HG-treated HK2 cells (Figure 2d).

### 3.3. The Impacts of NQO1 on the Expression of Markers of Renal Damages and Autophagy in HG-Treated HK2 Cells

**Figure 3:**
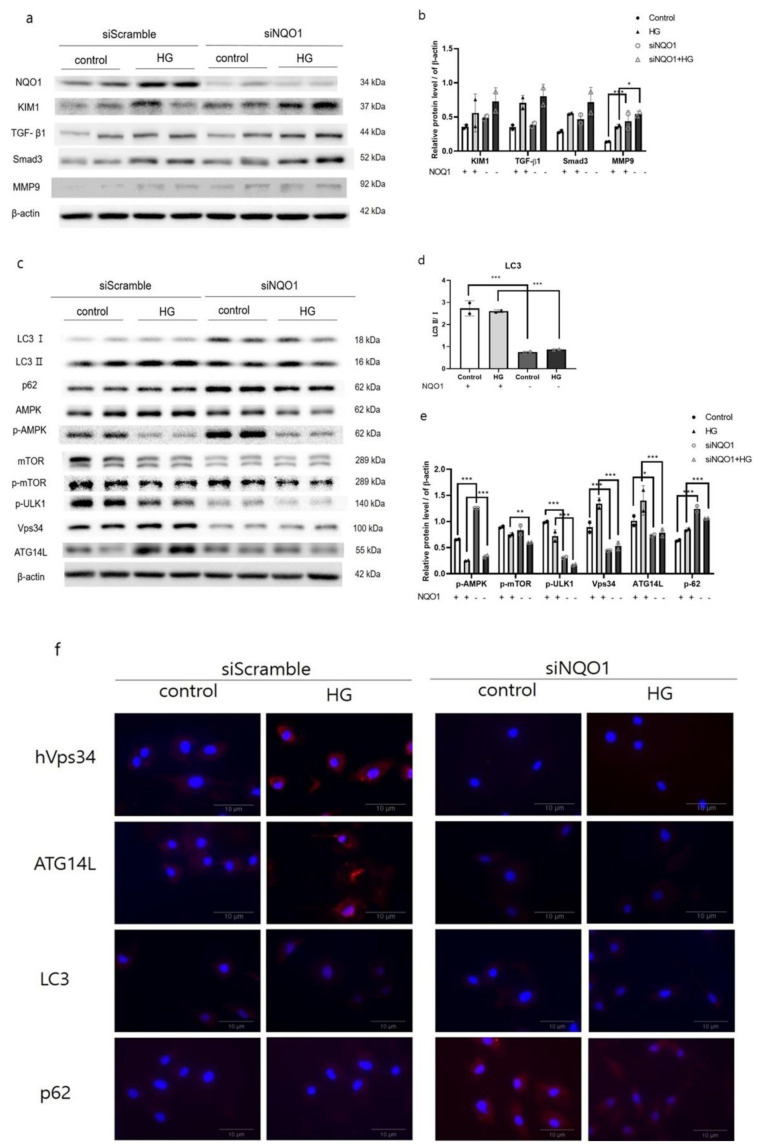
*NQO1* silencing aggravated kidney damages and dysregulated autophagy-related proteins in HG-treated HK2 cells. After siControl or si*NQO1* transfection, cells were incubated with vehicle of HG (25 mM) for 24 h. Total cell lysates were analyzed using western blot. Conversion ratio of LC3Ⅱ/Ⅰ (**d**). The expression intensities of kidney damages (**a**) and autophagy-related proteins (**c**) were normalized to those of β-actin (**b**,**e**). Values are expressed as means ± SEM of triplicate experiments, * *p* < 0.05, ** *p* < 0.01, *** *p* < 0.001 compared with wild-type. Cell nuclei were visualized by 6-diamino-2 phenylindole (DAPI; Blue) and the expressions of ATG14L, Vps34, LC3-Ⅱ, and p62 were visualized by AlexaFlour594 conjugate (Red) at 400× magnification (scale bar = 10 μm) (**f**).

**Figure 4:**
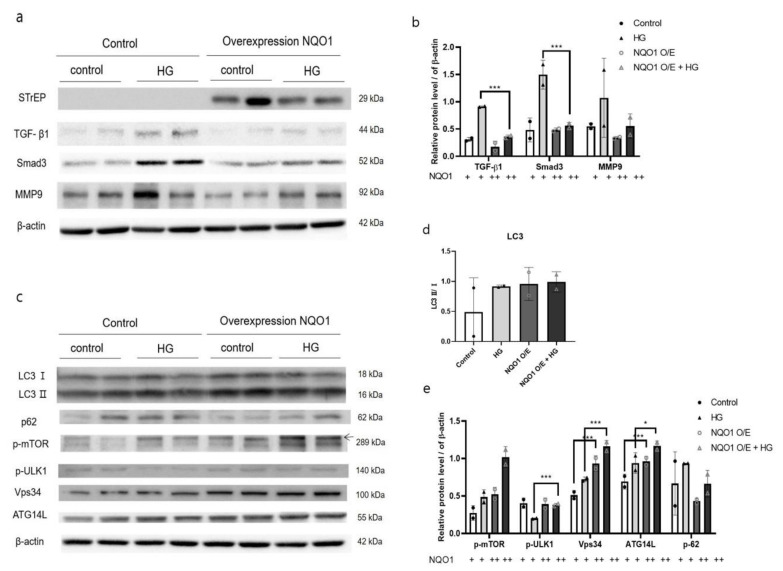
*NQO1* overexpression induces the autophagy and reduces kidney damages in HG-treated HK2 cells. After transfection with *NQO1* overexpression plasmids (Strep-tag pEXPR-IBA 105-NQO1), all cells were incubated for 24 h. Total cell lysates were analyzed using western blot. The expression intensities of kidney damages (**a**) and autophagy-related proteins (**c**) were normalized to those of β-actin (**b**,**d**,**e**). Values are expressed as means ± SEM of triplicate experiments, * *p* < 0.05, *** *p* < 0.001 compared with wild-type.

To investigate the specific role of NQO1 in the development of renal damages during DN, we transfected HK2 cells with siRNA targeting human *NQO1* or a *NQO1* overexpression plasmids (Strep-tag pEXPR-IBA 105-*NQO1*). To assess the effectiveness of transfection, we carried out western blot analysis, which confirmed the reduced expression of *NQO1* in transfected- HK2 cells (Figure 3a and Figure 4a).

Notably, HG treatment led to increased expression of KIM1 with increased TGF-β1, Smad3, and MMP9 in both si*NQO1*- and siControl-transfected HK2 cells with respect to untreated cells. Moreover, the expressions of all proteins were increased in si*NQO1*-transfected cells compared with their respective control. Especially, the expression of MMP9 was significantly (<0.001, <0.05) increased in si*NQO1*-transfected cell compare to their counterpart (Figure 3a,b).

On the other hand, in *NQO1*-overexpressing cells the basal expression of TGF-β1, Smad3, and MMP9 was reduced with respect to their control. In addition, upon HG treatment, the expression of these proteins was reduced to less than 50% of their level in respective control (Figure 4a,b).

Furthermore, the expression of LC3-Ⅱ was significantly reduced (*p* < 0.001) in *NQO1*-knockdown cells compared with cells transfected with a scrambled siRNA (Figure 3c,d). Conversely, the expressions of p62 significantly increased (*p* < 0.001) in siNQO1-transfected cells. Interestingly, the levels of p-ULK1, Vps34, and ATG14L were significantly diminished (*p* < 0.001) in si*NQO1*-transfected cells (Figure 3). As shown by immunofluorescence, the number of siControl-transfected HK2 cells positively stained for Vps34 and ATG14L notably increased after HG treatment. However, the basal expressions of Vps34 and ATG14L was reduced in si*NQO1*-transfected control cells. In addition, HG-treated si*NQO1*-transfected HK2 cells did not display a significant increase in Vps34 and ATG14L expression. The number of LC3-Ⅱ positive stained were reduced, whereas the number of p62 positively stained were increased in si*NQO1*-transfected cell compared to siControl-transfected HK2 cells (Figure 3f).

In summary, HG treatment accelerated the autophagy flux in *NQO1*-overexpressing cells, as demonstrated by the increase in LC3-Ⅱ levels and the decrease in p62 levels (Figure 4b,d). Moreover, the expression of p-ULK1, Vps34, and ATG14L, playing an important role in autophagy induction and phagophore formation, was significantly upregulated (*p* < 0.001) in both untreated and HG-treated *NQO1*-overexpressing cells with respect to control HK2 cells. However, p-mTOR, a negative regulator of autophagy, was also significantly more expressed in *NQO1*-overexpressing cells in control cells (Figure 4)

### 3.4. Kidney Damages Exacerbated in Streptozotocin-Treated Nqo1 KO Mice In Vivo

**Figure 5:**
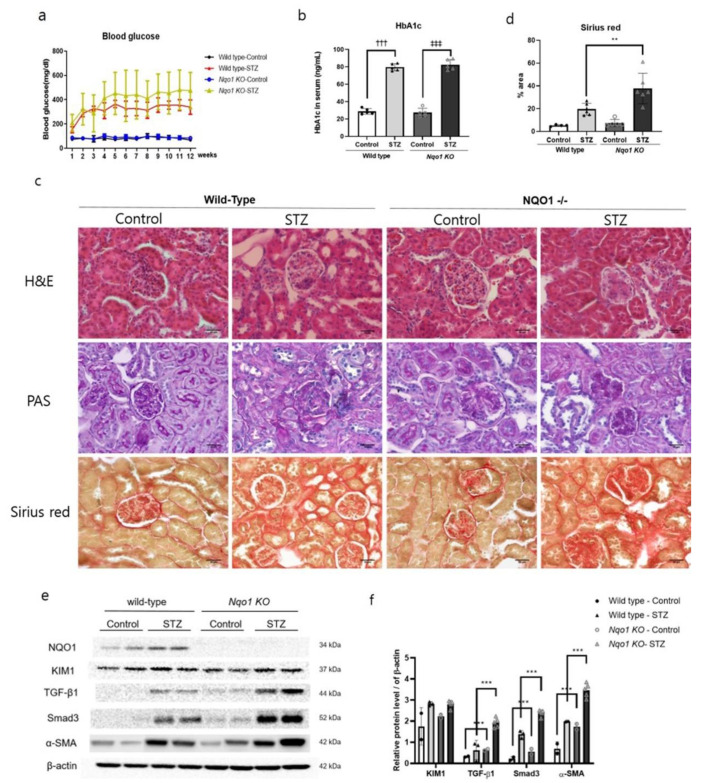
Increased renal damage in *Nqo1* KO mice in STZ-induced DN. C57BL/6 and *Nqo1* KO mice were sacrificed 12 week after STZ treatment. (*n* = 5/group) Blood glucose level (mg/dl) (**a**), serum HbA1c levels (**b**). Histopathology determined via hematoxylin and eosin (H&E), period acid-Schiff stain (PAS) and Sirius red stain at 400× magnification (scale bar = 30μm) (**c**). The % area of fibrosis was quantified (**d**). The expressions of kidney damages related proteins (**e**) were normalized to those of β-actin (**f**). Values are expressed as means ± SEM of triplicate experiments. ** *p* < 0.01, *** *p* < 0.001 compared with wild-type. ^†††^ *p* < 0.001 compared with wild type control. ^‡‡‡^ *p* < 0.001 compared with *Nqo1* KO control.

To confirm the results obtained with HK2 cells, *Nqo1* KO mice were subjected to STZ treatment to induce the DN. The body weights and kidney weights were shown in Table 1. As shown in Table 1, the relative kidney weight was increased in both of STZ-treated mice. Also, the blood glucose levels of STZ-treated mice were higher than those of control mice, especially, from the fourth week, when blood glucose exceeded 400 mg/dL in STZ-treated *Nqo1* KO mice. In addition, glycohemoglobin (HbA1c) levels were significantly increased (*p* < 0.001) in both STZ-treated groups (Figure 5a,b). Two indices of renal function, i.e., the serum levels of blood urea nitrogen (BUN) and creatinine, were enhanced in both of STZ-treated groups, prominently STZ-treated *Nqo1* KO mice (Table 2), together with increased expression of KIM1, a biological marker of proximal tubule damage (Figure 5e). In H&E and PAS staining, the kidneys of STZ-treated mice showed diffuse mesangium expansion with mesangial cell proliferation. In particular, STZ-treated wile-type mice displayed prominent mesangium expansions with early nodularity. On the other hand, STZ-treated *Nqo1* KO mice, showed accumulation of mesangial matrix forming Kimmelestiel-Wilson nodules, indicating grade 2b–3 glomerular changes associated to DN. Furthermore, in both STZ-treated groups, the tubular basement membranes were thickened and wrinkled with nucleus loss in proximal tubules. Especially in STZ-treated *Nqo1* KO mice, atrophic tubules, 25–50% of interstitial fibrosis, and some tubules containing cell debris with an interstitial fibrosis and tubular atrophy (IFTA) score of 2 were observed (Figure 5c).

In addition, the expressions of TGF-β1 and Smad3 showed a two-fold increase (*p* < 0.001) in STZ-treated wild-type and *Nqo1* mice with respect to their respective controls. Furthermore, α-SMA was markedly more expressed in both STZ-treated groups than in control groups, with an especially significant increase (*p* < 0.001) in *Nqo1* mice (Figure 5e,f). In addition, Sirius red staining showed that STZ treatment modulated the expression levels of collagen in the kidney. In fact, STZ-treated renal tissue showed increased accumulation of collagen in the glomerular interstitium and tubule-interstiti, especially significant (*p* < 0.001) in STZ-treated *Nqo1* KO mice rather than in STZ-treated wild-type mice. All together, these results demonstrated that *Nqo1* deficiency impaired autophagy by blocking induction and formation of the phagophore. In addition, renal fibrosis progressed further in STZ-treated *Nqo1* KO mice.

### 3.5. Nqo1 Deficiency Dysregulates Autophagy in STZ Induced DN

**Figure 6:**
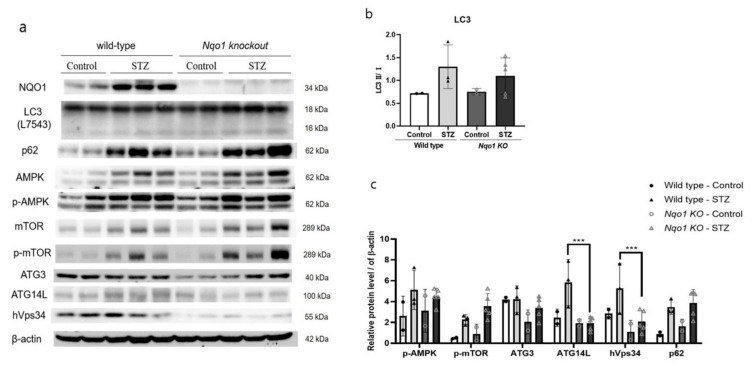
*NQO1* dysregulated autophagy initiation related proteins in STZ-induced DN. C57BL/6 and *Nqo1* KO mice were sacrificed 12 week after STZ treatment. (*n* = 5/group) Homogenized tissues were analyzed using western blot. The expression intensities of autophagy-related proteins (**a**) were normalized to those of β-actin (**b**,**c**). Values are expressed as means ± SEM of triplicate experiments, *** *p* < 0.001 compared with wild-type.

The expressions of p-AMPK and p-mTOR increased in both STZ-treated groups. However, in contrast with the in-vitro result, the expressions of LC3-Ⅱ were increased in both STZ-treated groups. The p62 was also more expressed in both STZ-treated groups. On the other hand, interestingly, consistently with the in-vitro results, the basal expression of ATG14L and Vps34 was significantly reduced (*p* < 0.001) in *Nqo1* KO mice (Figure 6).

## 4. Discussion

Resulting from the deterioration of chronic renal disease, DN, mainly arises from imbalanced activation of several molecular mechanisms that regulate oxidative stress, inflammation, autophagy and cell death [38]. However, piles of evidence point at oxidative stress as a driving factor of autophagy in DN, which was found to have an important renoprotective function in different types of renal cells [23,36]. Although many studies focused on the roles of oxidative stress and autophagy in DN pathogenesis, the implications of defective redox and autophagy signaling in DN progression are not yet fully elucidated.

In the present study, the impact of *NQO1* on the DN pathogenesis was explored by modulating *NQO1* expression by using in-vitro and in-vivo models.

NQO1 is a well-known cytoprotective protein that mediates detoxification of reactive metabolites [39]. NRF2 and NQO1 protein expressions were upregulated under the oxidative stress to prevent or slow the progression of oxidative damages [3]. HG stress was reported to induce oxidative stress and leads to enhanced accumulation of TGF-β1, a key mediator of renal fibrosis pathogenesis that promotes de-differentiation of proximal tubules, in HK2 cells [39,40]. In this study, HG stress showed enhanced expressions of NQO1 and its upstream signaling, NRF2, in HK2 cells. Moreover, increased expression of KIM1, an indicator of renal damage, was accompanied an obvious increased in the expression of fibrosis-related markers, including MMP9, TGF-β1, and α-SMA. Under the HG stress, the present enhanced NQO1 expression might be enhanced to counteract HG induced oxidative stress and worsen renal fibrosis.

In addition, HG treatment initiated the autophagosome formation in HK2 cells, as confirmed by increased expression of autophagosome elongation-related markers. However, interestingly, expression of p-AMPK and p-mTOR upstream autophagy signal, in high glucose treated HK2 cells were not in general. Autophagy is promoted by phosphorylation of AMPK, which is an energy sensor that regulates cellular metabolism, and suppressed by activation of mTOR which is a cell-growth regulator that integrates growth factor and nutrient signals [41]. Nevertheless, several studies have reported that under HG conditions SIRT-1 and endoplasmic reticulum stress can induce autophagy through an AMPK/mTOR independent pathway [23,42,43]. In line with these previous studies, we found that HG-driven damage induced autophagy independently of AMPK/mTOR. It seems worth further researching on this part.

Various studies have reported that autophagy suppresses renal fibrosis and may provide a pro-survival role [26,27,28,30,31]. In the present study, HG treatment aggravate renal fibrosis by suppressing the autophagy flux confirmed by p62 accumulation in HK2 cells. After 3-MA treatment, the autophagy formation was blocked accumulating p62 expression in HG-treated HK2 cells. When the autophagy flux was blocked by 3-MA treatment, the expression of renal fibrosis signal, TGF-β1, was increased. These changes confirmed by collagen and fibronectin deposition by confocal microscopy. However, with rapamycin treatment, which induces autophagy by inhibiting mTOR activity [44], these results were contrary to the previous results. These results indicated that HG stress aggravated DN progression by suppressing autophagy flux.

To further evaluate the role of NQO1 in DN progression under HG stress, *NQO1* was silenced or overexpressed in HK2 cells. *NQO1*-silenced cells showed markedly enhanced HG-induced renal cell injury, as indicated by KIM1 expression. Moreover, HG induced renal damage due to the *NQO1* absence confirmed by the evidently enhanced expression of pro-fibrotic proteins including TGF-β1, Smad3, and MMP9. TGF-β1 is known to drive renal fibrosis via down-stream Smad-dependent signaling and is involved in epithelial-mesenchymal transition and in the regulation of ECM turnover through the induction of pro-fibrotic molecules, including α-SMA, collagen 1 and MMP9 [16,42,45]. Moreover, Smad3 plays a critical role in mediating fibrosis during DN, through the activation of myofibroblasts, stimulation of excessive ECM production and inhibition of ECM degradation [27]. In addition, HG-induced cell damages were markedly reduced in *NQO1* overexpressing cells when compared to siControl-transfected cells.

Oxidative stress mediated regulation of autophagy in various organs. It is generally reported that a loss of oxidative stress increased the expression of LC3-Ⅱ by decreased mTOR signaling [46]. In breast cancer cells, NRF2 negatively regulate autophagy formation [47]. On the other hand in intervertebral disc disease, NRF2 KO mouse reduces autophagy [48]. In a previous study, a lack of *NQO1* exacerbated oxidative stress, leading to enhanced autophagy in cisplatin-treated ACHN cells [34]. In this context, we speculated that increased HG-induced damage in the absence of *NQO1* would result in an elevated autophagy flux. [49]. However, surprisingly, si*NQO1*-transfected cells displayed evidently lower levels of LC3-Ⅱ together with higher p62 expression, indicating dysregulated initiation and completion of the autophagy process. The amount of LC3 at a certain time point does not indicate autophagy flux. It is important to measure the amount of LC3-Ⅱ converted from LC3-Ⅰ [50]. p62 which is a receptor for cargo to be degraded by autophagy. When a series of autophagy process occurs, it is called autophagy flux [49]. Moreover, *NQO1*-silenced cells showed an evidently lower expression of basal autophagy initiation-related signals such as p-ULK1, Vps34, and ATG14L, when compared to the si-Control-treated cells. This was also observed in untreated *NQO1*-silenced HK2 cells. Two protein complexes are involved in the initiation of autophagy: the class Ⅲ PI3K complex, consisting of Vps34, ATG14L, and ATG6/beclin 1; and the ATG/ULK1 complex [51,52]. The ULK complex guides the class Ⅲ PI3K complex to the phagophore assembly site and the class Ⅲ PI3K complex induces in autophagy and possibly phagophore formation [53,54]. Indeed, the suppression of Vps34 and ATG14L was confirmed by fluorescence staining in *NQO1*-silenced cells. Therefore, the dysregulation of autophagy in our experimental setup might be due, at least in part, to the substantial suppression of basal autophagy initiation-related signal expressions, which in turn autophagy process dysregulation. That is, absence of *NQO1* interferes with the normal functioning of the regulating autophagy mechanism resulting in side effects on active level. This hypothesis was also supported by the results of *NQO1* overexpression. In fact, untreated *NQO1*-overexpressing cells showed comparable basal levels of autophagy-related signals to those of untreated Control-transfected cells. Moreover, HG-induced cell damages were markedly reduced in *NQO1*-overexpressing cells when compared to si-Control-transfected cells, accompanied by an enhanced autophagy flux. These results indicated under the HG stress condition, NQO1 deficiency aggravated renal fibrosis by dysregulating Vps34/ATG14L autophagy initiation complex. NQO1 might play a key role in the regulation of autophagy in HK2 cells and in fibrosis remission under HG stress conditions.

To confirm the impact of defective NQO1 in the promotion of autophagy and fibrosis during DN, a further in vivo study was performed with a *Nqo1* KO mice model. Although, these in no BUN and creatinine statistically significant between *Nqo1* KO and wild-type mice, exacerbation of renal tissue damage due to the lack of *Nqo1* was clearly observed by histopathology. Indeed, mesangial expansion with nodularity was observed in both STZ-treated groups; in particularly, STZ-treated *Nqo1* KO mice displayed accumulation of Kimmelestiel-Wilson nodules together with progression of fibrosis. Furthermore, tubular atrophy and tubular basement membrane thickening were advanced in STZ-treated *Nqo1* KO mice.

In our in-vivo study, the expression pattern of upstream autophagy signaling molecules such as p-AMPK and p-mTOR, did not exactly match with that from the in vitro study with HK2 cells, because various cell types, including podocytes, mesangial cells, and glomerular endothelial cells, reside in the kidney tissue. However, similarly to the in-vitro study, the *Nqo1* KO mice showed a dysregulated autophagy process with suppression of signals related to autophagy initiation and autophagosome elongation. That is, absence of *Nqo1* interferes with the normal functioning of the regulating autophagy mechanism resulting in side effects on active level. The expressions of these proteins was accompanied by significantly increased renal tissue damage and fibrosis-related signals after STZ treatment. In particular, the expression of the pro-fibrotic markers, Smad3, α-SMA, and collagen, was especially enhanced in STZ-treated *Nqo1* KO mice, implying the progress of interstitial fibrosis implying leading to end-stage kidney disease. The upregulation of renal NQO1 and its related signals was found to alleviate STZ-induced diabetic renal damage by reducing oxidative damages and suppressing the TGF-β1/Smad3 pathway [55]. Consistent with these reports, our results have demonstrated that absence of NQO1 showed increased expression of profibrotic markers. This implies that renal fibrosis is aggravated in absence of NQO1.

In our in-vitro study, in si*NQO1*-transfected cells, the cell damages and fibrosis were induced under the HG stress condition accompanied by a reduced autophagy flux. However, HG-induced cell damages were markedly reduced in *NQO1*-overexpressing cells when compared to Control-transfected cells, accompanied by an enhanced autophagy flux. In our in vivo study, the *Nqo1* KO mice showed a dysregulated autophagy process with suppression of signals related to autophagy initiation and elongation. Suppression of autophagy-related proteins was accompanied by aggravation of renal tissue damage and fibrosis-related signals levels after STZ treatment in *Nqo1* KO mice.

## 5. Conclusions

**Figure 7:**
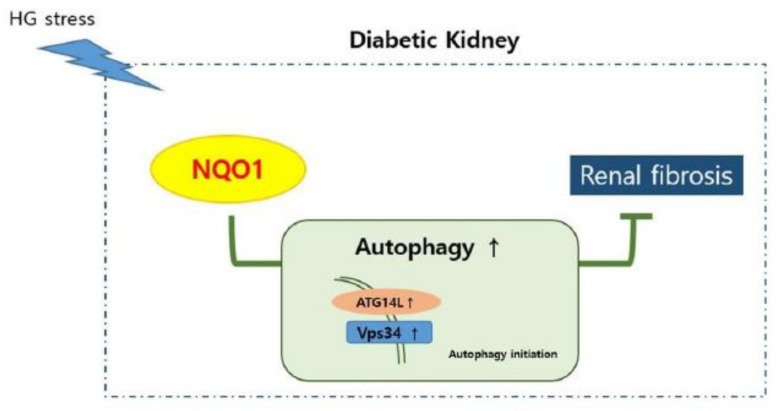
Schematic representation of NQO1, ATG14L, Vps34, autophagy, and renal fibrosis in diabetic nephropathy. In diabetic nephropathy, under the stress, NQO1 is induced. In the absence of NQO1, autophagy initiation was dysregulated, resulting suppressing autophagy. In turn, ensuring of NQO1 pathway and autophagy flux reduces renal fibrosis.

In this study, the impact of NQO1 expression on autophagy and fibrosis progression during DN was evaluated. In summary, the absence of NQO1 dysregulated the autophagy flux with the aggravation of renal damage. The present study has demonstrated that defective expression of the redox signal NQO1 leads to the dysregulation of autophagy and results in exacerbation of DN-related renal fibrosis (Figure 7). Taken together, our results indicate that ensuring the activation of the *NQO1* pathway and of the autophagy flux can be a practical therapeutic strategy against DN-associated fibrosis progression.

## Figures and Tables

**Table 1:** Body weights and relative kidney weights in STZ-treated DN mouse models.

	Body Weights (g)	Relative Kidney Weights (g)
WT Control	37.3 ± 0.7	1.2 ± 3.0
WT STZ	42.2 ± 1.8	1.5 ±0.1
*Nqo1* KO Control	25.0 ± 0.2	1.2 ± 1.4
*Nqo1* KO STZ	51.4 ± 5.0 ^‡‡^	1.7 ± 0.0

^‡‡^*p* < 0.001 compared with *Nqo1* KO Control.

**Table 2:** BUN and Creatinine diameters in STZ-treated DN mouse models.

	BUN (g/dL)	Creatinine (mg/dL)
WT Control	29.8 ± 2.3	0.2 ± 0.0
WT STZ	41.5 ± 2.0	0.3 ± 0.0
*Nqo1* KO Control	25.1 ± 0.2	0.1 ± 0.0
*Nqo1* KO STZ	51.9 ± 6.1 ^‡‡^	0.5 ± 0.2

^‡‡^*p* < 0.001 compared with *Nqo1* KO Control.

## Data Availability

The datasets generated and analyzed during the current study are available from the corresponding authors on request.

## Supplementary Materials

The following are available online at https://www.mdpi.com/2076-3921/10/2/333/s1.
