# Supplementary material for: NQO1 Deficiency Aggravates Renal Injury by Dysregulating Vps34/ATG14L Complex during Autophagy Initiation in Diabetic Nephropathy
Source: Antioxidants (Basel). 2021 Feb 23;10(2):333. doi: 10.3390/antiox10020333 (PMC7926338; doi:10.3390/antiox10020333)
Supplement: Supplementary file 1 [file antioxidants-10-00333-s001.pdf]

Suppliment 1

	Antibody	Dilution ratio	Company	State	Country	catalog number
1	anti-LC3	1:1000	Abcam	Cambrige	UK	ab7543
2	anti-p62	1:1000	Cell Signaling Technology	MA	USA	#5114S
3	anti-mTOR	1:1000	Gene Tex	TX	USA	GTX101557
4	anti-AMPK	1:1000	Cell Signaling Technology	MA	USA	#2532S
5	anti-ATG7	1:1000	Santa Cruz	TX	USA	SC33211
6	anti-ATG14L	1:1000	Abcam	Cambrige	UK	ab139727
7	anti-NQO1	1:1000	Santa Cruz	TX	USA	SC16464
8	anti-KIM1	1:1000	Gene Tex	TX	USA	GTX12016
9	anti-TGF-β1	1:1000	Abcam	Cambrige	UK	ab64175
10	anti-Smad3	1:1000	Santa Cruz	TX	USA	SC101154
11	anti-α-SMA	1:1000	Abcam	Cambrige	UK	ab5694
12	anti-matrix metallopeptidase 9 (MMP9)	1:1000	Abcam	Cambrige	UK	ab38898
13	anti-β-actin	1:5000	Abcam	Cambrige	UK	ab8229
14	anti-Vps34	1:1000	Abcam	Cambrige	UK	ab124905

Suppliment 2

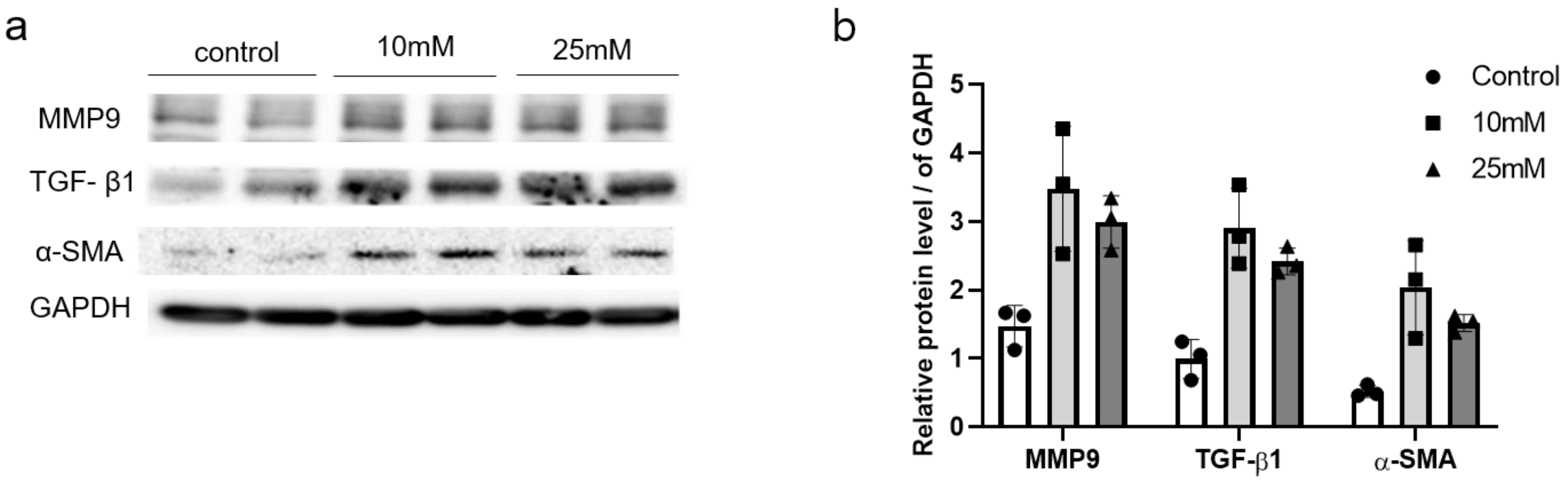

Suppliment 2. TGF-β1, the primary factor driving fibrosis, and matrix metallopeptidase 9 (MMP9), catalyzing the release of active TGF-β1, were expressed two times more in HG-treated cells than in the control. Finally, the expression of α-SMA contributing to the slow contractile properties of myofibroblasts, was increased upon treatment with a 10 and 25 mM glucose concentration

Suppliment 3

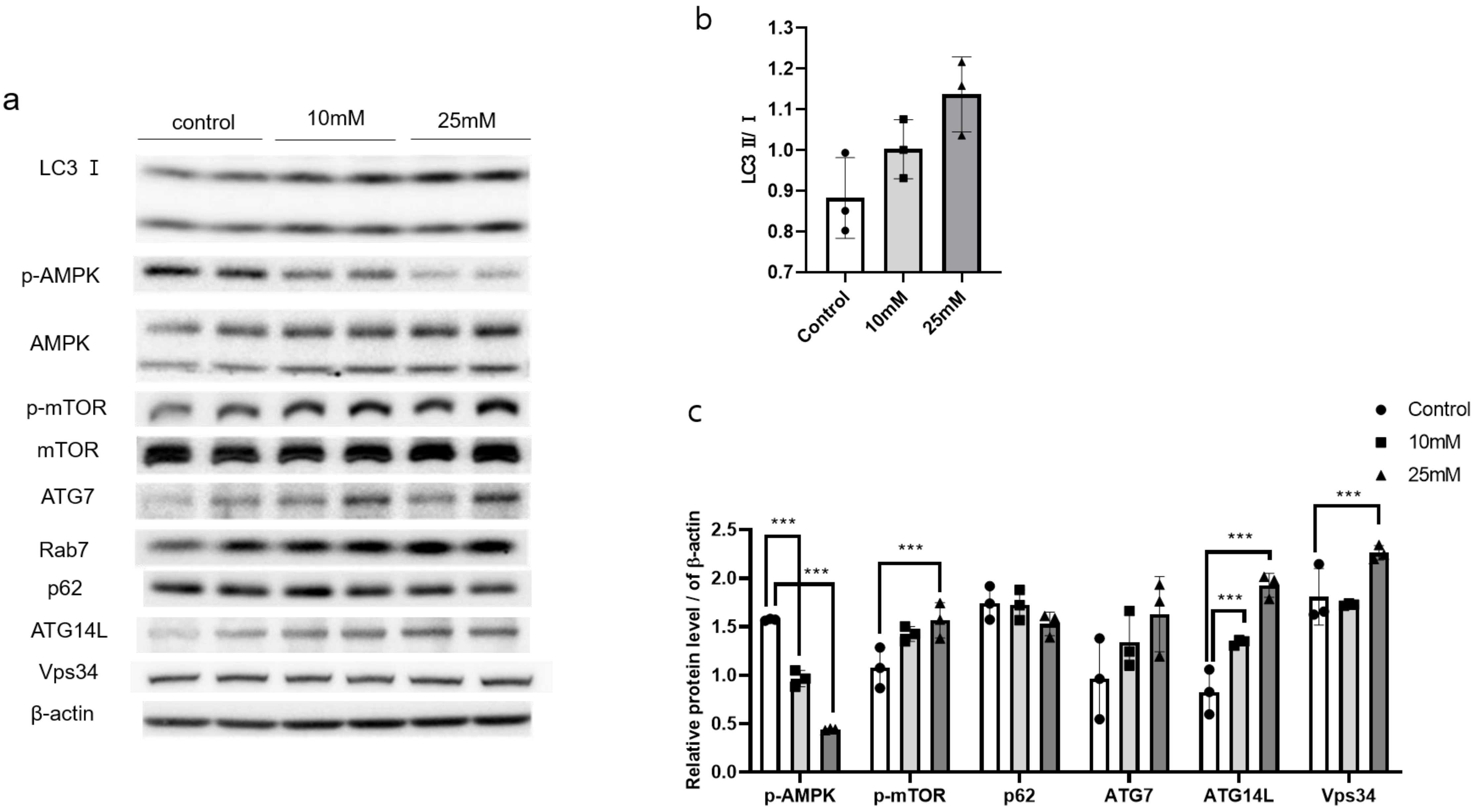

Suppliment 3. The expression of LC3-Ⅱ was gradually increased while that of p62 was reduced in glucose treated cell. Moreover, the relative protein level of p-AMPK, a promoter of autophagy, was progressively significantly reduced ($P < 0.001$), while that of p-mTOR, an inhibitor of autophagy, gradually increased with higher glucose concentration.

Furthermore, the expression of ATG14L and Vps34, which are needed to form the class Ⅲ PI3 K complex, was significantly upregulated by HG ($P < 0.001$)
